# First Cultivation and Characterization of *Mycobacterium ulcerans* from the Environment

**DOI:** 10.1371/journal.pntd.0000178

**Published:** 2008-03-26

**Authors:** Françoise Portaels, Wayne M. Meyers, Anthony Ablordey, António G. Castro, Karim Chemlal, Pim de Rijk, Pierre Elsen, Krista Fissette, Alexandra G. Fraga, Richard Lee, Engy Mahrous, Pamela L. C. Small, Pieter Stragier, Egídio Torrado, Anita Van Aerde, Manuel T. Silva, Jorge Pedrosa

**Affiliations:** 1 Institute of Tropical Medicine, Antwerpen, Belgium; 2 Armed Forces Institute of Pathology, Washington, D.C., United States of America; 3 Noguchi Memorial Institute for Medical Research, College of Health Sciences, University of Ghana, Legon, Accra, Ghana; 4 Life and Health Sciences Research Institute (ICVS), School of Health Sciences, University of Minho, Braga, Portugal; 5 Department of Microbiology, University of Tennessee, Knoxville, Tennessee, United States of America; 6 Institute for Molecular and Cell Biology, University of Porto, Porto, Portugal; Institut Pasteur, France

## Abstract

**Background:**

*Mycobacterium ulcerans* disease, or Buruli ulcer (BU), is an indolent, necrotizing infection of skin, subcutaneous tissue and, occasionally, bones. It is the third most common human mycobacteriosis worldwide, after tuberculosis and leprosy. There is evidence that *M. ulcerans* is an environmental pathogen transmitted to humans from aquatic niches; however, well-characterized pure cultures of *M. ulcerans* from the environment have never been reported. Here we present details of the isolation and characterization of an *M. ulcerans* strain (00-1441) obtained from an aquatic Hemiptera (common name Water Strider, *Gerris* sp.) from Benin.

**Methodology/Principal Findings:**

One culture from a homogenate of a *Gerris* sp. in BACTEC became positive for IS*2404*, an insertion sequence with more than 200 copies in *M. ulcerans*. A pure culture of *M. ulcerans* 00-1441 was obtained on Löwenstein-Jensen medium after inoculation of BACTEC culture in mouse footpads followed by two other mouse footpad passages. The phenotypic characteristics of 00-1441 were identical to those of African *M. ulcerans*, including production of mycolactone A/B. The nucleotide sequence of the 5′ end of 16S rRNA gene of 00-1441 was 100% identical to *M. ulcerans* and *M. marinum*, and the sequence of the 3′ end was identical to that of the African type except for a single nucleotide substitution at position 1317. This mutation in *M. ulcerans* was recently discovered in BU patients living in the same geographic area. Various genotyping methods confirmed that strain 00-1441 has a profile identical to that of the predominant African type. Strain 00-1441 produced severe progressive infection and disease in mouse footpads with involvement of bone.

**Conclusion:**

Strain 00-1441 represents the first genetically and phenotypically identified strain of *M. ulcerans* isolated in pure culture from the environment. This isolation supports the concept that the agent of BU is a human pathogen with an environmental niche.

## Author Summary

*Mycobacterium ulcerans* infection, or Buruli ulcer, is the third most common mycobacteriosis of humans worldwide, after tuberculosis and leprosy. Buruli ulcer is a neglected, devastating, necrotizing disease, sometimes producing massive, disfiguring ulcers, with huge social impact. Buruli ulcer occurs predominantly in impoverished, humid, tropical, rural areas of Africa, where the incidence has been increasing, surpassing tuberculosis and leprosy in some regions. Besides being a disease of the poor, Buruli ulcer is a poverty-promoting chronic infectious disease. There is strong evidence that *M. ulcerans* is not transmitted person to person but is an environmental pathogen transmitted to humans from its aquatic niches. However, until now *M. ulcerans* has not been isolated in pure culture from environmental sources. This manuscript describes the first isolation, to our knowledge, of *M. ulcerans* in pure culture from an environmental source. This strain, which is highly virulent for mice, has microbiological features typical of African strains of *M. ulcerans* and was isolated from an aquatic insect from a Buruli ulcer–endemic area in Benin, West Africa. Our findings support the concept that *M. ulcerans* is a pathogen of humans with an aquatic environmental niche and will have positive consequences for the control of this neglected and socially important tropical disease.

## Introduction

Buruli ulcer (BU), the third most common mycobacteriosis in humans after tuberculosis and leprosy is an indolent, necrotizing disease of skin, subcutaneous tissue and, occasionally, bones [1]. BU has emerged in recent times as an increasingly important cause of morbidity around the world, and has been reported in 30 countries, mostly in tropical areas [2]. This disease is caused by *Mycobacterium ulcerans* which is peculiar among pathogenic mycobacteria because it produces a potent necrotizing exotoxin, mycolactone, which is a major virulence factor [3].

Although incompletely understood, the epidemiology of BU strongly associates the disease with wetlands and especially slow-flowing or stagnant water [4]–[6]. Indeed, there is indirect evidence that *M. ulcerans* is an environmental pathogen transmitted to humans from its aquatic niches; however, modes of transmission are unclear [7]. The initial hypothesis that predatory aquatic insects, including Naucoridae and Belostomatidae, were involved in transmission [8] was later reinforced by reports that the salivary glands of Naucoris were colonized by *M. ulcerans* when fed on infected grubs, and that bites of infected Naucoris transmitted the pathogen to mice [9]. The observation that non-infected humans exposed to aquatic environments in BU endemic areas have higher titers of antibodies to salivary proteins of Naucoridae and Belostomatidae than BU patients in the same areas [10] shows that these water bugs bite humans in natural settings. However, Naucoridae and Belostomatidae are carnivorous insects that normally feed on other aquatic insects, snails, and small fish and only bite humans incidentally [11]. Thus, the significance of biting by *M. ulcerans*-colonized aquatic insects in the transmission of BU to humans is unknown, and other forms of transmission, including skin trauma, have been considered [12].

Since the discovery of IS*2404* [13], an insertion sequence with more than 200 copies in *M. ulcerans* [14], multiple studies have detected IS*2404* in environmental aquatic samples, indicating that *M. ulcerans* is probably present in such samples, and supporting the concept that the etiologic agent of BU is an environmental pathogen. IS*2404* was found in samples of water and detritus from swamps in Australia [15],[16],[17], and in aquatic plants [18], insects (Belostomatidae, Naucoridae, Hydrophilidae), crustaceans and mollusks (*Bulinus* sp. and *Planorbis* sp.), and fish (including *Tilapia* sp.) in western tropical Africa [8],[9],[18],[19],[20]. More recently IS*2404* was detected in mosquitos in Australia [21]. However, the recent discovery of IS*2404* in aquatic mycobacteria other than *M. ulcerans* requires re-evaluation of the use of IS*2404* PCR for the detection of *M. ulcerans* DNA in the environment [22],[23] and emphasizes the importance of the isolation of *M. ulcerans* from environmental sources.

Numerous extensive studies have failed to isolate *M. ulcerans* in pure culture from the environment, even in highly endemic areas of BU, e.g. in Uganda [24], the Democratic Republic of Congo [4],[5],[25] and West Africa [19].

Two cultures from salivary glands of wild aquatic insects (Naucoridae) collected in BU endemic areas of Côte d'Ivoire were positive for IS*2404* and were considered to be related to *M. ulcerans*; however, no phenotypic characteristics of these isolates were reported other than their virulence for mice [9]. In 2004, Marsollier *et al.* obtained IS*2404* PCR positive cultures from two samples of aquatic plants collected in a BU endemic area of Côte d'Ivoire [18]. One IS*2404* positive BACTEC culture inoculated into mice revealed infection compatible with *M. ulcerans*. The culture was, however, contaminated by *Mycobacterium szulgai* and *M. ulcerans* could not be obtained in pure culture even after passages through mice.

As briefly reported previously [26], a pure culture of *M. ulcerans* (isolate 00-1441) was obtained from an aquatic insect from Benin. In that report no description was given of the methods employed for the isolation of *M. ulcerans* 00-1441 and of the phenotypic and genetic characteristics of the isolate. Here we present the detailed results of the isolation and characterization of strain 00-1441, establishing that this mycobacterium is an African type of *M. ulcerans* with high virulence for mice. Strain 00-1441 represents the first well characterized *M. ulcerans* strain isolated in pure culture from an environmental source.

## Materials and Methods

### Environmental specimens

#### Collection and in vitro culture

Four aquatic specimens collected in a BU endemic area of Benin and one in Togo that were part of a larger study on the frequency of mycobacteria in the environment (Portaels *et al.*, manuscript in preparation) were cultured in vitro and inoculated into mouse footpads. The specimens were transported from the field to the laboratory in sterile tubes at 4°C and kept frozen until they were identified by an entomologist.

Types of specimens collected and their origins are indicated in Table 1.

**Table 1:** Ziehl-Neelsen staining, culture and PCR results for 5 aquatic specimens collected in Buruli ulcer endemic regions of Benin and Togo

N° specimen	Family (genus)	District (Country)	ZN	IS*2404* PCR	Culture on LJ	Growth in BACTEC		
						GI reached 999 after (weeks)	ZN	IS*2404* PCR
98–449	Lymnaeidae *(Lymnaea* sp.*)*	Afagnan (Togo)	-	-	*M. nonchronogenicum*	2	+	-
98–457	Pleidae *(Plea* sp.*)*	Zagnanado (Benin)	-	-	*M. fortuitum*	2	+	-
98–447	Naucoridae *(Naucoris* sp.*)*	Ouinhi (Benin)	-	-	*M. gordonae*	2	+	+
97–1455	Belostomatidae *(Appasus* sp.*)*	Ouinhi (Benin)	-	-	Contaminated	2	+	+
98–443	Gerridae *(Gerris* sp.*)*	Zagnanado (Benin)	-	-	*M. gordonae*	2	+	+

GI, growth index.

The specimens were thoroughly diced with sterile disposable scalpels in a sterile mortar. They were further homogenized with a sterile pestle and suspended in 2 ml phosphate buffered saline (PBS) (Oxoid, Hampshire, England; pH = 7.3±0.2). The mortars and pestles were used only for environmental specimens. Decontamination was performed by treatment of the suspensions for 15 minutes with an equal volume of aqueous 2.0% sodium hydroxide (NaOH) containing sodium citrate (1.45% final concentration) and N-acetyl-l-cysteine (0.5% final concentration). The suspensions were centrifuged for 20 minutes at 3,800 g and fractions of the resulting sediments were used for Ziehl-Neelsen staining (ZN), culture and PCR.

Primary cultures were performed at 32°C by inoculating the sediment on Löwenstein-Jensen (LJ) solid medium and in BACTEC 12B broth (Becton Dickinson Microbiology Systems, Sparks, MD, USA) supplemented with PANTA and 1.25% egg yolk [27]. Growth index (GI) was measured weekly for 3 months with a BACTEC 460 TB instrument (Becton Dickinson, Sparks, MD, USA). When the GI reached 999, ZN was performed, and BACTEC cultures were tested by IS*2404* PCR [28]. Inoculated LJ tubes were incubated for 12 months and observed every 2 weeks.

#### PCR on decontaminated specimens and BACTEC cultures

DNA was extracted from decontaminated specimens and BACTEC positive cultures as previously described with minor modifications [29]. Briefly, 250 µl suspensions were treated with equal volumes of lysis buffer L6 (5 M GuSCN, 5 0 mM Tris, pH 6.4, 22 mM EDTA, 2% Triton X-100) followed by 50 µl of proteinase K (20 mg/ml). This mixture was incubated overnight at 60°C with gentle shaking. To capture DNA, 40 µl of diatomaceous earth stock solution (10 g diatomaceous earth obtained from Sigma Aldrich Chemie GmbH, Steinheim, Germany) in 50 ml of H_2_O containing 500 µl of 37% (wt/vol) HCl was added to the suspension and placed in a shaker incubator at 37°C for 2 hours to avoid sedimentation of the diatomaceous earth.

The pellets were washed with 900 µl of L2 buffer (5 M GuSCN, 50 mM Tris, pH 6.4) [29] followed by 900 µl of 70% ethanol and 900 µl of acetone. The pellet was then dried at 55°C and resuspended in 90 µl TE (10 mM Tris, 1 mM EDTA, pH 8). Tubes were centrifuged and 50 µl of the DNA extract transferred to a new tube.

PCR reaction: A nested PCR was performed targeting the insertion sequence element IS*2404* with four primers: PGP1 (5′-AGGGCAGCGCGGTGATACGG-3′), PGP2 (5′-CAGTGGATTGGTGCCGATCGT-3′), PGP3 (5′-GGCGCAGATCAACTTCGCGGT-3′) and PGP4 (5′-CTGCGTGGTGCTTTACGCGC-3′) (GenBank accession n° U38540), [13].

The following procedures were employed:

For the first PCR run, 5 µl of the DNA extract was added to 45 µl PCR reaction mixture containing 20 pmol of each primer (PgP1 and PgP2), 1U of Taq DNA polymerase (Roche Molecular Systems, Brussels, Belgium), 200 µM concentrations of each deoxyribonucleotide triphosphate, 1.5 M MgCl_2_, 0.1% X-100, and 10 mM Tris–HCl (pH 8.4) and overlaid with mineral oil. Cycling was as follows: denaturation at 94°C for 5 min; amplification for 40 cycles at 94°C for 45 sec, 64°C for 45 sec and 72°C for 45 sec and a final extension at 72°C for 10 min.

For the second PCR run, 0.25 µl of the first-run product was amplified in a 25 µl reaction mixture with primers PgP3 and PgP4. Cycling conditions were similar to the first run except that amplification was reduced to 25 cycles. Amplified DNA (7 µl) was then submitted to electrophoresis in 2% agarose gel and detected by ethidium bromide staining and UV transillumination.

#### Mouse footpad inoculation

The three IS*2404* PCR positive BACTEC cultures (Table 1) were inoculated (0.03 ml) into the left hind footpad of three 8 week old female mice (strain NMRI).

Mice were sacrificed after 6, 9 or 12 months (Table 2). Entire feet were placed into 10% formalin for histopathological analysis, or were used for microbiological analysis as described previously [30]. For specimens 97–1455 and 98–443, mouse footpad suspensions (harvested after 9 months) were also passaged (P1) into 3 other mice. A second passage (P2) was done from P1 after 12 months. No passage was performed for specimen 98–447 because only one mouse survived for 9 months and the entire foot of this mouse was used for histopathologic analysis.

**Table 2:** Results of inoculation of mouse footpads with BACTEC cultures of 2 environmental specimens

Specimen	Passage in mouse (1)	Mouse n°	Death/Sacrifice (after)	Macroscopic aspect	ZN	Culture on LJ	Histopathology
98–447	P1	1	Death (1 month)	normal	NT	NT	NT
		2	Death (1 month)	normal	NT	NT	NT
		3	Sacrifice (9 months)	normal	NT	NT	Large numbers of beaded AFB in granulomas
97–1455	P1	1	Death (1 month)	normal	NT	NT	NT
		2	Sacrifice (9 months)	normal	NT	NT	Clumps of AFB in necrotic area.
		3	Sacrifice (9 months)	normal	+	99–2832	Active stage of BU
						(*M.* sp. IS*2404* positive)	NT
	P2	1	Death (1 month)	normal	NT	NT	NT
		2	Death (1 month)	normal	NT	NT	NT
		3	Sacrifice (12 months)	normal	-	contaminated	NT
	P3	1	Sacrifice (6 months)	normal	-	-	NT
		2	Sacrifice (6 months)	normal	-	-	NT
		3	Sacrifice (12 months)	normal	-	-	NT
98–443	P1	1	Death (1 month)	normal	NT	NT	NT
		2	Sacrifice (9 months)	normal	NT	NT	Large numbers of beaded AFB in granuloma
		3	Sacrifice (9 months)	normal	-	-	NT
	P2	1	Death (6 months)	normal	NT	NT	NT
		2	Sacrifice (12 months)	normal	NT	NT	Minimal nonspecific inflammation
		3	Sacrifice (12 months)	normal	-	-	NT
	P3	1	Sacrifice (6 months)	normal	-	00-1441	NT
						(*M. ulcerans)*	
		2	Sacrifice (6 months)	normal	NT	NT	Mild nonspecific inflammation
		3	Sacrifice (12 months)	normal	NT	NT	No change

(1) P1 = first passage from BACTEC to mouse footpads.

P2 = second passage from mouse footpad (P1) to mouse footpads.

P3 = third passage from mouse footpad (P2) to mouse footpads.

#### Histopathologic analyses

Histopathologic analyses of mice feet were made at the indicated times after inoculation of positive BACTEC vials and, in some cases, after passage of inoculated footpads to other mice. The entire feet were intact at this stage. Specimens were decalcified with a solution containing 4% concentrated hydrochloric acid and 4% concentrated formic acid in distilled water or with a commercial solution (Thermo, Shandon TBD-1 Rapid Decalcifier, Chesire, UK) for a period of 45 min. Multiple longitudinal sections 1.5–2 mm thick were cut, processed routinely and sectioned at 4 µm. Sections were stained by hematoxylin-eosin, ZN, Grocott's methenamine-silver and Brown-Hopps Gram methods [31].

#### Identification of the mycobacteria

Mycobacteria cultivated directly from the aquatic specimens and the *M. ulcerans* isolate 00-1441 cultivated from a mouse footpad (see Table 2) were identified as described previously [32].

### Analysis of isolate 00-1441

#### 16S rRNA gene sequencing and phylogenetic analysis

The isolate 00-1441 was also identified by partial analysis of the 3′ end region as described previously [33] as well as the 5′ end region of the 16S rRNA gene by Eurogentec (Liège, Belgium) with an automated nucleic acid sequencer (Applied Biosystems, Foster City, CA, USA).

#### Mycolactone extraction and analysis

Acid soluble lipid containing mycolactones were extracted from *M. ulcerans* strains 1615 and 00-1441 with chloroform-methanol 2∶1 followed by back extraction with ice-cold acetone as previously described [34]. Lipids were resolved by silica thin-layer chromatography using a chloroform-methanol-water (90∶10∶1) solvent system and visualized by charring with anisaldehyde in 10% sulfuric acid.

Mass spectrometric (MS) analysis of the mycolactone extracts was performed as previously described [34]. Ten milligrams of the dried acetone soluble lipid (ASL) extract were re-suspended in 1 milliliter of methanol and subsequently filtered prior to MS analysis. Using a Cole Palmer 74900 series syringe pump, the methanolic extract was perfused into an ion trap ESI Bruker-Esquire mass spectrometer at dry temperature of 300°C, gas flow of 5 liters/min and nebulizer pressure of 15 lb/in^2^.

#### Mouse footpad inoculation

The present study was conducted under the guidelines and approval of the Research Ethics Committee of the Life and Health Sciences Research Institute (Braga, Portugal).

Isolate 00-1441 was grown on LJ medium at 32°C for approximately 2 months, recovered from LJ slants, diluted in PBS to a final mass concentration of 1 mg/ml, and vortexed vigorously using 2-mm glass beads. In all the experiments, the number of acid-fast bacilli (AFB) in each inoculum was determined by the method of Shepard and McRae [35], using ZN staining (Merck, Darmstadt, Germany). The suspensions revealed more than 90% viable bacilli as assessed with the LIVE/DEAD BacLight Kit (Molecular Probes, Leiden, The Netherlands).

Eight-week-old female BALB/c or NMRI mice were obtained from Charles River Laboratories (Barcelona, Spain) and were housed in specific pathogen-free conditions with food and water *ad libitum*. Both strains of mice were infected in the left hind footpad with 0.03 ml of a suspension containing 5.4 log_10_ AFB of *M. ulcerans* 00-1441.

As an index of lesion development, footpad swelling was measured over time with a caliper. Bacterial proliferation was evaluated in footpad homogenates of infected mice at selected time points, as previously described [30],[36]. Histological analysis of the feet was carried out as described above.

#### Infection of murine bone marrow-derived macrophages

Bone marrow-derived macrophages (BMDM) from BALB/c mice were used as mouse primary macrophages and were prepared as previously described [36]. Briefly, both femurs were removed under aseptic conditions. Bones were flushed with cold Hank's Balanced Salt Solution (HBSS, Gibco, Paisley, UK). The resulting cell suspension was centrifuged and resuspended in Dulbecco's Modified Eagle's Medium (DMEM, Gibco) supplemented with 10 mM HEPES (Sigma, St. Louis, MO), 1 mM sodium pyruvate (Gibco), 10 mM glutamine (Gibco), 10% of heat-inactivated fetal bovine serum (Sigma) and 10% L929 cell-conditioned medium (Complete DMEM [cDMEM]). To remove fibroblasts or differentiated macrophages, the cells were cultured at 37°C in a 5% CO_2_ atmosphere for a period of four hours in cell culture dishes (Nunc, Naperville, IL) with cDMEM. The non-adherent cells were collected with warm HBSS, centrifuged and distributed in 24-well plates at a density of 5×10^5^ cells/well and incubated at 37°C in a 5% CO_2_ atmosphere. L929 cell-conditioned medium was added 4 days after seeding and medium was renewed on the seventh day. After 10 days in culture, cells were completely differentiated into macrophages. Twelve hours before infection, macrophages were incubated at 32°C in a 5% CO_2_ atmosphere and maintained at 32°C until the end of the experimental infection.

For macrophage infectivity assays bacterial suspensions were prepared as described above and further diluted in cDMEM to obtain the selected multiplicity of infection (MOI) of 1∶1 (bacteria/macrophage ratio). Cells were incubated for 4 hours at 32°C in a 5% CO_2_ atmosphere and then washed with warm HBSS to remove non-internalized bacteria and re-incubated in cDMEM for eight days.

#### Genotyping methods

Genotyping methods developed to analyze the diversity among *M. ulcerans* and *M. marinum* strains from different geographical areas were applied to isolate 00-1441. IS*2404* restriction fragment length polymorphism (RFLP) and PCR restriction profile analysis (PRPA) were performed as described previously [37],[38].

Mycobacterial Interspersed Repetitive Units (MIRU) Variable Number Tandem Repeat (VNTR) analysis was also used to investigate the MIRU-VNTR profile of 00-1441 as previously described [39],[40].

## Results

### Environmental specimens

Results of ZN staining, culture and PCR studies for the 5 aquatic specimens are shown in Table 1. Table 2 shows the results of the mouse footpad inoculation with the BACTEC suspensions (98–447, 97–1455 and 98–443) that were positive by IS*2404* PCR after inoculation with the aquatic specimens.

***Specimen 98–447:*** Histopathologic analysis of one mouse sacrificed after 9 months revealed a few well formed granulomas with minimal necrosis around blood vessels, nerves and in muscle. There were large numbers of beaded AFB in the granulomas.

***Specimen 97–1455:*** Of the three mice inoculated with this BACTEC culture (P1 in Table 2), two were sacrificed 9 months after inoculation. The histopathologic analysis of the footpad of one mouse showed marked necrosis with a mild granulomatous response, inflammation of periosteum and many large clumps of AFB in necrotic areas. The footpad homogenate of the third mouse was positive for AFB and the culture on LJ was positive for mycobacteria (isolate 99–2832) but was lost due to contamination by nonacid-fast bacteria; however, PCR performed on the contaminated culture was positive for IS*2404*. This mouse footpad was inoculated in vitro and passaged twice into two groups of three mouse footpads. The second (P2 in Table 2) and third passages (P3 in Table 2) were negative for AFB by ZN staining and by culture.

***Specimen 98–443:*** This homogenate of a *Gerris* sp. aquatic insect ultimately produced the *M. ulcerans* isolate 00-1441 after culture in BACTEC (positive for IS*2404*) inoculated in mouse footpads (P1) and followed by two other mouse footpad passages (P2 and P3).

Following the first mouse inoculation (P1), one animal died after 1 month and the other two were sacrificed 9 months after inoculation. Histopathologic evaluation of the footpad of one of these mice showed granulomatous changes with minimal necrosis around blood vessels and nerves. There were large numbers of scattered, short, beaded AFB in the granuloma. ZN stain and culture were negative for the footpad of the third mouse inoculated in vitro. The suspension obtained from the third mouse was used to reinoculate 3 other mice (P2). One P2 mouse died after 6 months and the other two were sacrificed 12 months later. The histopathologic study of one footpad showed minimal nonspecific inflammation. The other mouse footpad was negative for AFB and by culture and was used for a third passage (P3) into 3 mice. Two of the P3 mice were sacrificed after 6 months. One footpad used for histopathologic study showed nonspecific inflammation. The other footpad was ZN-negative but gave a positive culture on LJ (5 colonies) after 2 months incubation at 32°C. The isolate (00-1441) was further analyzed and identified as *M. ulcerans* (see below).

The remaining P3 mouse, sacrificed after one year, did not reveal any histopathologic changes.

### Characterization of isolate 00-1441

#### Phenotypic characterization

The phenotypic characteristics of *M. ulcerans* isolate 00-1441 and those of some *M. ulcerans* geographic subgroups are given in Table 3. Isolate 00-1441 has the same phenotypic characteristics as *M. ulcerans* strains belonging to the African subgroup [33], i.e., it was scotochromogenic, did not grow on LJ containing 250 µg/ml hydroxylamine and was acid phosphatase positive.

**Table 3:** Phenotypic characteristics of isolate 00-1441 and *M. ulcerans* geographic subgroups

	Geographic subgroups of *M. ulcerans* ^(1)^						
	AF	AUS	MEX	S. AM.	CHI	JAP	00-1441
N° of tested strains			2	4	1	1	1
Pigmentation in the dark	+(1)	-	-	+	-	+	+
Pigmentation in the light	+(1)	-	-	+	-	+	+
Growth at 37°C	-	-	-	-	-	-	-
Growth on peptone agar	-	-	-	-	-	-	-
Growth in presence of:							
Isoniazid (10 µg/ml)	+	M	+	-	-	-	+
Thiophene-2-carboxylic hydrazide (2 µg/ml)	+	+	+	+	+	-	+
Hydroxylamine (250 µg/ml)	-	+	+	M	+	+	-
p-Nitrobenzoate (500 µg/ml)	-	-	+	-	+/−	-	-
NaCl 5%	-	-	-	-	-	-	-
Enzymatic properties							
Catalase, >45 mm of foam	-	-	-	-	-	+	-
Tween 80 hydrolysis (10 days)	-	-	-	-	-	-	-
Urease activity	-	-	-	-	-	-	-
Niacin production	-	-	-	-	-	-	-
Nitrate reduction	-	-	-	-	-	-	-
Acid phosphatase activity	M	-	-	-	-	-	+
Sequencing results (Portaels *et al.,* 1996)	Type 1	Type 2	Type 3	*M. marinum*	*M. shinshuense*	*M. shinshuense*	Type 1

AF = African, AUS = Australian, S.AM = South American, CHI = Chinese, JAP = Japanese subgroup (*M. shinshuense*); (1) light yellow pigment; +: >85% of strains positive; -: <15% of strains positive; M: 50 to 85% of strains positive; F: 15 to 49% of strains positive.

#### 16S rRNA gene sequencing and phylogenetic analysis

The 5′ end of 16S rDNA sequence was 100% identical to that of *M. ulcerans* [41].

Sequencing results of the 3′ end of 16s rDNA for 00-1441 and the different *M. ulcerans* types are given in Table 3.

Isolate 00-1441 was characterized by a G at position 1248 shared by *M. ulcerans* type 1, type 2, type 3, and *Mycobacterium shinshuense*, a C at position 1289 typical of *M. ulcerans* type 1 and type 2 and CTTT at positions 1450–1452 unique to *M. ulcerans* type 1 [33]. However, a point mutation was found at position 1317, with a T instead of a C typical for all *M. ulcerans* types and *Mycobacterium marinum*.

#### Mycolactone analysis

**Figure 1:**
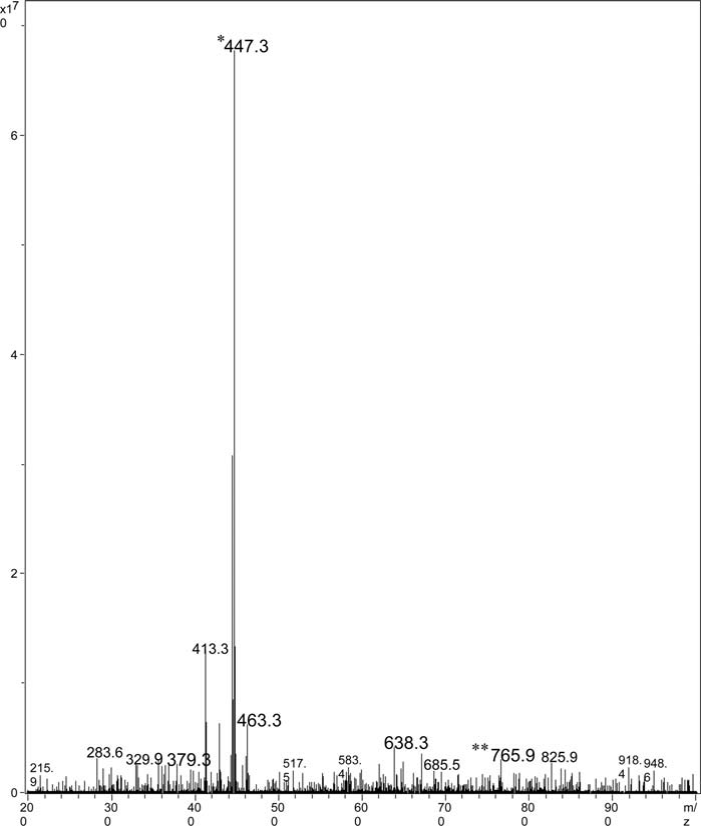
Mass spectroscopic analysis of ASL of *M. ulcerans* 00-1441 showing the hydrolysis product of mycolactone A/B at m/z 447.3 (*). The intact mycolactone can be identified at lower intensity at m/z 765.9 (**).

**Figure 2:**
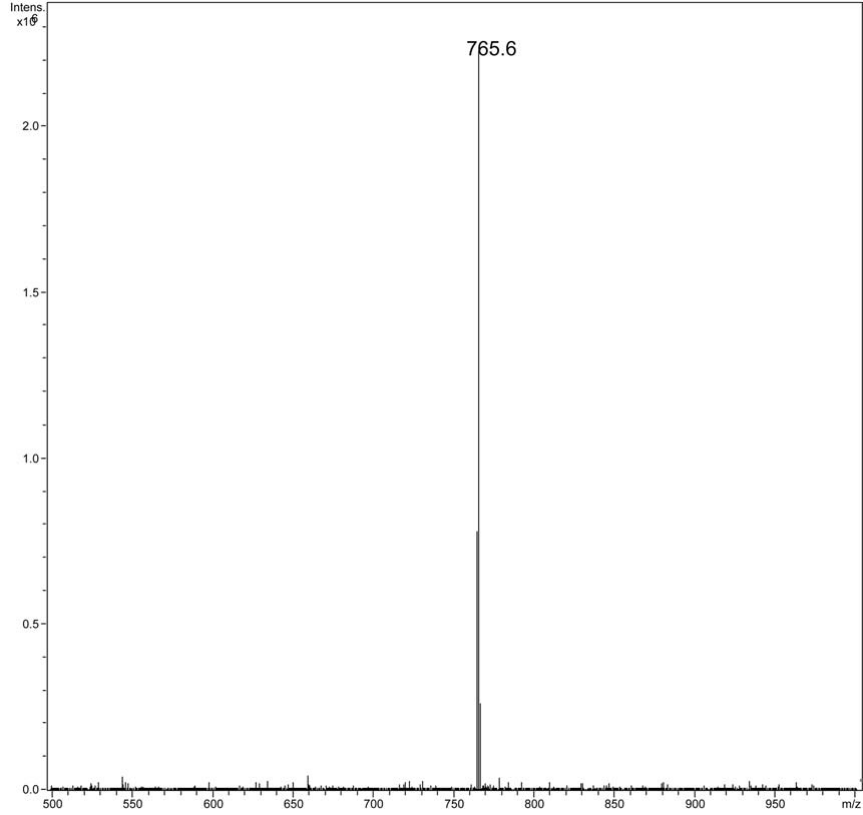
Mass spectroscopic analysis of the ASL of *M. ulcerans* 00-1441 showing the mycolactone A/B sodium adduct at m/z 765.6. (In this case an ion trap was set to select for positive ions in the range m/z 755–775.)

**Figure 3:**
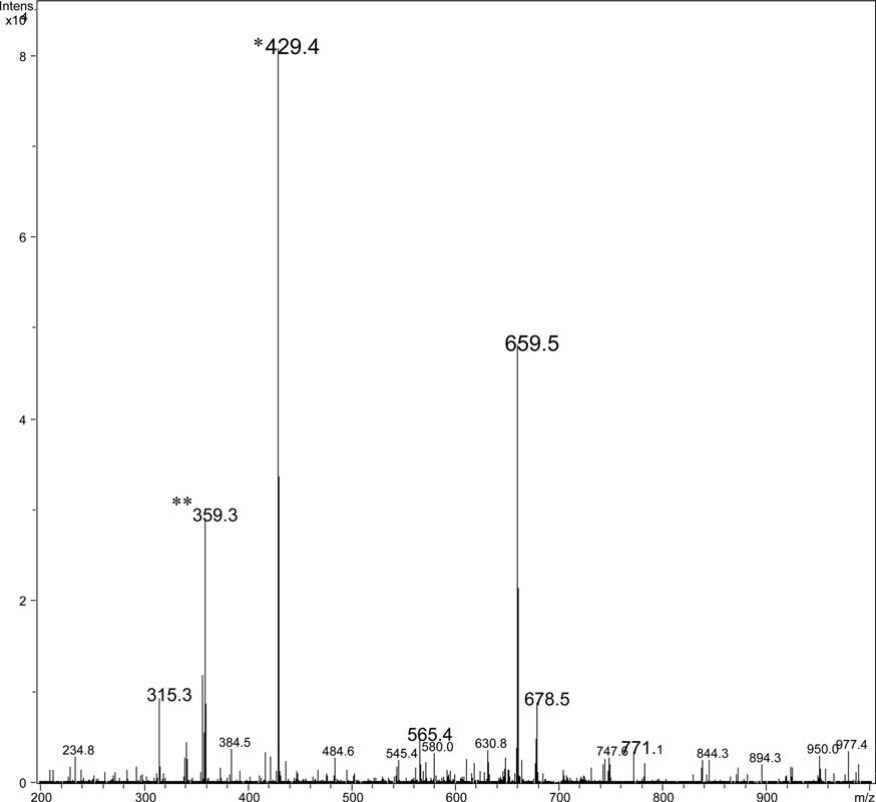
MS/MS fragmentation pattern of the mycolactone A/B ion at m/z765.6. The two main characteristic fragments are those of the macrolide ring m/z = 429.4 (*) and the side chain at m/z = 359.3 (**).

Thin-layer chromatography of ASLs showed that isolate 00-1441 produces mycolactone A/B as a major lipid species (data not shown). Mass spectroscopy provided definitive evidence for mycolactone production as evidenced by sodium adducts at 765.9, representing the intact mycolactone molecule, and the hydrolysis product showing the core ion at 447.3 (Fig. 1). Enrichment for the intact ion using ion trap for positive ions between m/z 755–775 revealed a major peak consistent with intact mycolactone A/B (Fig. 2). Fragmentation pattern of this species following MS-MS demonstrated the presence of the characteristic mycolactone fragmentation pattern with core mycolactone at 429.4 and fatty acid side-chain at 359.3 (Fig. 3).

### Infection of murine bone marrow macrophages

**Figure 4:**
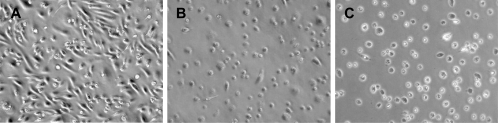
Cytotoxic activity of *M. ulcerans* 00-1441 to BMDM. BMDM were infected with *M. ulcerans* 00-1441 at an MOI of 1∶1. Macrophages were photographed by phase-contrast microscopy at 4 h (A), 4 days (B), and 6 days (C) postinfection. Macrophage cell rounding, shrinkage and detachment are present at days 4 and 6 post-infection.

As previously described for virulent *M. ulcerans* strains [36], isolate 00-1441 showed cytotoxic activity against BMDM infected at an MOI 1∶1 as deduced at day 4 post-inoculation from the occurrence of mycolactone-associated cytopathic signs [42] namely, cell rounding, shrinkage and detachment of the macrophages (Fig. 4).

#### Mouse footpad inoculation

**Figure 5:**
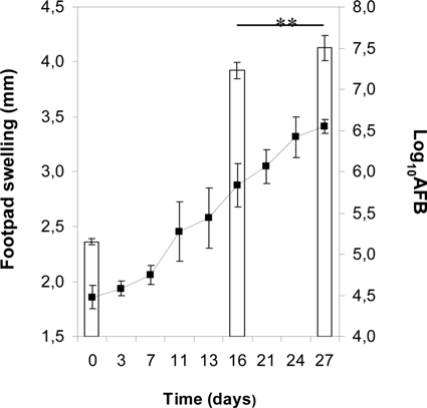
Footpad swelling and bacterial proliferation during infection with isolate 00-1441. BALB/c mice were infected subcutaneously in the footpad with 5.2 log^10^ AFB of *M. ulcerans* 00-1441. The degree of pathologic changes was assessed by measurement of footpad swelling (▪) (n = 8). The proliferation of bacilli in the footpad was determined by counting AFB (open bars) of footpad homogenates (n = 5). Significant differences were performed using Student's t test (**, p≤0.01).

Footpads of three NMRI mice showed swelling beginning 7 days after inoculation of 00-1441. Isolate 00-1441 was also virulent for BALB/c mice. The proliferation of bacilli, assessed by AFB counts, and the level of pathologic changes, evaluated by footpad swelling and emergence of ulceration, were monitored throughout the experimental period of infection of BALB/c mice footpads. As shown in Fig. 2, swelling became apparent during the second week of infection; ulceration was observed after the fourth week post-inoculation. AFB counts in footpad homogenates increased significantly from 5.15 log_10_ to 7.50 log_10_ between days 0 and 27 post-inoculation (Fig. 5).

**Figure 6:**
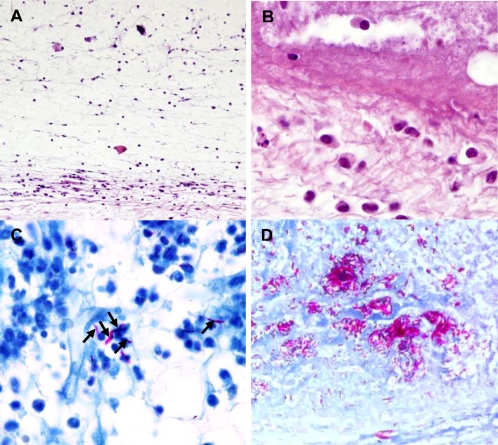
Histologic sections of mouse footpads infected with *M. ulcerans* 00-1441. BALB/c (A–C) and NMRI (D) mice were infected subcutaneously with 5.4 log^10^ *M. ulcerans* 00-1441. At different time points the footpads were harvested and processed for histologic analysis. Dermal edema could be found by the second week of infection (A), along with a necrotic center surrounded by both chronic and acute inflammatory infiltrates (B) (hematoxylin-eosin stained sections). After 4 weeks of infection, bacilli were observed co-localized with cells (C) and in large clumps in the necrotic center of the lesion (D) (Ziehl-Neelsen stained sections).

Observation of serial footpad sections of BALB/c mice showed an acute neutrophilic inflammatory response in the subcutaneous tissue early after infection with 00-1441 (data not shown). In the course of the second week of infection, evidence of dermal edema was found (Fig. 6A) along with a mixed inflammatory infiltrate containing mononuclear cells and neutrophils (Fig. 6A and B), surrounding the necrotic center of the lesion (Fig. 6B). By weeks 3 to 4, the necrotic focus expanded progressively, invading healthy tissue and numerous AFB were observed in areas co-localizing with leukocytes, predominantly of the mononuclear type (Fig. 6C). In more advanced stages of infection, and concurring with ulceration, the subcutaneous tissue of BALB/c and NMRI mice revealed extensive necrotic acellular areas with clumps of free bacilli (Fig. 6D).

**Figure 7:**
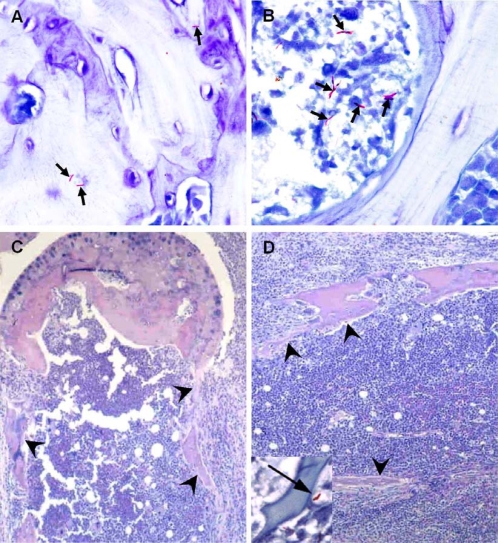
Ziehl-Neelsen staining of bone tissue of mice infected with *M. ulcerans* 00-1441 (A, B and inset of D, arrows). NMRI mice were infected subcutaneously in the footpad with 5.4 log^10^ *M. ulcerans* 00-1441. At 6 weeks post-infection, bacilli were found both in the bone tissue and in the bone marrow (A, B and inset in D, arrows). Hemotoxylin-eosin staining of C and D reveals extensive destruction of bone. Outlines of dead cortical bone spicules are seen in C and D (arrowheads). Marrow is necrotic and replaced by chronic inflammation.

An important histopathological finding regarding footpad infection by strain 00-1441 in NMRI and BALB/c mice was the presence of AFB in the bones (Fig. 7A) and bone marrow (Fig. 7B) of the feet. Extensive destruction of the bone was observed with erosion of the cortex and replacement of marrow by inflammatory cells (Fig. 7C and D).

These results indicate that *M. ulcerans* strain 00-1441 is highly virulent for mice.

#### Genotyping results

The IS*2404* RFLP banding pattern of 00-1441 was identical to that of African *M. ulcerans* isolates [37]. Using PRPA, 00-1441 also yielded a profile similar to that of African *M. ulcerans* isolates [38].

Comparison of MIRU-VNTR profile based on 6 loci showed that 00-1441 has a typical Atlantic African genotype [40].

## Discussion

The prevailing concept that BU is associated with wetlands, especially slow-flowing or stagnant water, implies that *M. ulcerans* is an environmental pathogen transmitted to humans from particular aquatic niches. Historically, the presence of *M. ulcerans* in aquatic samples, including water, mud, aquatic plants, aquatic insects, aquatic mollusks, crustacea and small fish, has been inferred from the detection by PCR of the insertion sequence IS*2404*, highly represented in the genome of *M. ulcerans* [14]. All previous attempts to isolate fully characterized *M. ulcerans* from environmental samples, however, have failed, and recent evidence [22] indicates that IS*2404* positivity alone is inadequate to establish the presence of *M. ulcerans* in environmental samples.

*M. ulcerans* 00-1441, isolated from a Hemiptera (Water Strider, *Gerris* sp.) collected from a swamp in a BU endemic region (Zagnanado, Benin), represents the first fully characterized culture of the agent of BU from an environmental source. Isolate 00-1441 was identified as *M. ulcerans* by the following criteria:

1. Phenotypic characteristics are identical to those of *M. ulcerans* strains from Africa [26];
2. Nucleotide sequence of the 5′ end of 16S rRNA gene has 100% identity with *M. ulcerans* and *M. marinum*;
3. Nucleotide sequence of the 3′ end of 16S rRNA gene is identical to that of *M. ulcerans* (African type) [33] except for a single nucleotide substitution at position 1317;
4. Identical profile with the predominant African type for the genotypic IS*2404*-RFLP, PRPA assays [37],[38], and MIRU-VNTR [40];
5. Identical mycolactone A/B to that produced by *M. ulcerans* strains from Africa [34];
6. Progressive infection and disease produced in mouse footpads with lesions showing histopathological features similar to those described in *M. ulcerans*-infected mice [36] and humans [43].

Additionally, 00-1441 had been previously found to have a mycolate profile pattern similar to that of *M. ulcerans*, with three types of mycolates, α-, methoxy-, and ketomycolates [26]. Moreover, 00-1441 and the predominant African type share identical profiles for IS*2404*-Mtb2 PCR [44], and microsatellite VNTR analysis [45].

Based on nucleotide substitutions at the 3′ end 16S rRNA gene [33], isolate 00-1441 is an *M. ulcerans* type 1 strain (an African type). The mutation found at position 1317 (a T instead of a C) has not been found previously. Indeed the 3′ end of the 16S rRNA gene of all *M. ulcerans* strains analyzed in 1996 [33] and of all other mycobacterial species has a C at position 1317 [Blast search on the nucleotide collection (nr/nt) database (NCBI) using the nucleotide sequence of the 3′end 16S rRNA gene (nt 1244-1461) of *M. ulcerans* type 1]. In a recent study on 75 *M. ulcerans* isolates from 17 different countries including 10 African countries (Angola, Benin, Cameroon, Congo-Brazzaville, Côte d'Ivoire, Democratic Republic of Congo, Ghana, Nigeria, Togo and Uganda), a few isolates from patients originating from the Zou and Ouémé valleys in Benin presented a T instead of a C at position 1317 (Portaels *et al.*, in preparation). Interestingly, strain 00-1441 was isolated from the region (Zou Department) where some of these patients lived.

The aquatic specimens analyzed in the present study likely contained very few mycobacteria since direct smear examination after decontamination was negative for all specimens and primary cultures positive for mycobacteria other than *M. ulcerans* (Table 1) produced only 1 to 3 colonies. Moreover, despite the very high sensitivity of the IS*2404* PCR [14], detection of IS*2404* in the decontaminated specimens was negative indicating that less than 10 mycobacterial cells were present in each suspension [28]. Culture in BACTEC allowed multiplication of the rare mycobacteria present in the inocula since three of the five BACTEC positive cultures were positive by IS*2404* PCR.

Our previous attempts to detect *M. ulcerans* in more than 1000 environmental specimens by culture have revealed numerous environmental mycobacteria belonging to species frequently cultivated from the environment [5]. However, other than the results of Marsollier *et al.* [9],[18] and the present study, all attempts to culture *M. ulcerans* from the environment have failed. As discussed elsewhere [19], there are several possible explanations for the difficulty in culturing *M. ulcerans* from environmental specimens, namely: (i) These specimens are heavily contaminated with other microorganisms, [5],[13],[18],[24]. This is primarily because the generation time of *M. ulcerans* is longer than that of most other slow-growing mycobacteria that are abundant in the environment [18],[19]. In the present study, successive passages in mice of BACTEC cultures may have eliminated mouse avirulent environmental mycobacteria [30] co-existing in the specimen, allowing multiplication of *M. ulcerans*. (ii) All decontamination methods currently available for the isolation of *M. ulcerans* from contaminated environmental specimens have a detrimental impact on the viability of this pathogen [27]. (iii) Since *M. ulcerans* is sensitive to elevated temperatures [46]–[47], temperature during transportation of environmental specimens to the laboratory is critical, particularly in tropical areas where ambient temperatures often exceed 32°C. (iv) As is the case in the present work, environmental specimens used in attempts to isolate *M. ulcerans* may contain very few bacilli. (v) Additionally, in the environment *M. ulcerans* may be living in a viable but nonculturable (VBNC) state. This state may represent a survival adaptation to overcome adverse conditions, but the organism retains secluded cultural viability and virulence capability [48],[49]. Most pathogenic bacteria of humans are known to enter the VBNC state, including those in aquatic environments [48],[50],[51]. The recuperation of culturability in bacteriological media by mycobacteria in the VBNC state may require a suitable resuscitation medium [52] and BACTEC may serve to resuscitate the VBNC *M. ulcerans.* Additional experiments are required to test for a VBNC state in environmental *M. ulcerans*.

**Figure 8:**
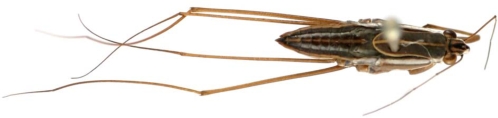
An example of *Afrotropical Gerridae*: *Limnogonus hypoleucus* (Gerstaecker). Photo: Jérome Constant, Department of Entomology, Royal Belgian Institute of Natural Sciences, Brussels, Belgium.

The strain analyzed in the present study was isolated from a Hemiptera (*Gerris* sp.). *Gerris* sp. belongs to the worldwide family of the Gerridae. They are elongate insects with very long mid and hind legs (Fig. 8). The latter allow them to move rapidly on water surfaces to catch their preys. They live on the surface of quiet waters and are unable to walk on the ground, but can fly from one pond or river to another [53].

Several publications have suggested that Hemiptera (Naucoridae, Belostomatidae) may play a role in the transmission of BU to humans [1],[8],[9]. The successful cultivation of *M. ulcerans* from another family of aquatic Hemiptera (Gerridae) extends the range of hypothetical hemipteran transmitters. Like other aquatic Hemiptera, Gerridae are aggressive predators of other aquatic organisms such as insects and small fish. However, there are no reports of Gerridae biting humans (Dethier M, personal communication) and these insects may be only passive, incidental and transient reservoirs of *M. ulcerans* without an obvious role in the transmission of BU to humans or other mammals.

MS analysis of ASLs confirmed that isolate 00-1441 produced mycolactone A/B. The virulence of *M. ulcerans* is largely due to the presence of the toxic macrolide, mycolactone [3]. It is now recognized that there is a family of mycolactones produced by *M. ulcerans* and other related mycobacterial species. Each mycolactone has a distinct structure and mass. However, all isolates of *M. ulcerans* from Africa produce mycolactone A/B [34]. The demonstration of mycolactone A/B in Gerridae isolate 00-1441 presented here provides additional evidence that this strain is similar to virulent strains isolated from patients throughout West Africa.

Like other mycolactone A/B producing *M. ulcerans* strains [36], strain 00-1441 proliferates extensively in mouse footpads and produces intense footpad swelling. Moreover, in previous mouse footpad inoculation studies on 11 isolates of *M. ulcerans* from patients in Benin, 5 of which were from bones of patients with *M. ulcerans* osteomyelitis [54], no changes were noted in the bones of the mice feet (Portaels F and Meyers WM, unpublished observations). However, feet of NMRI and BALB/c mice inoculated with 00-1441 showed striking destruction of bone. These data regarding mouse infection suggest that strain 00-1441 is highly virulent for mice. Additional experiments in mice and ex vivo are required to compare the virulence of strains sharing the same “T” for “C” 16S rRNA gene polymorphism at position 1317 and identically treated i.e., after several passages in mice. Such experiments are underway and will be presented in another publication.

**Figure 9:**
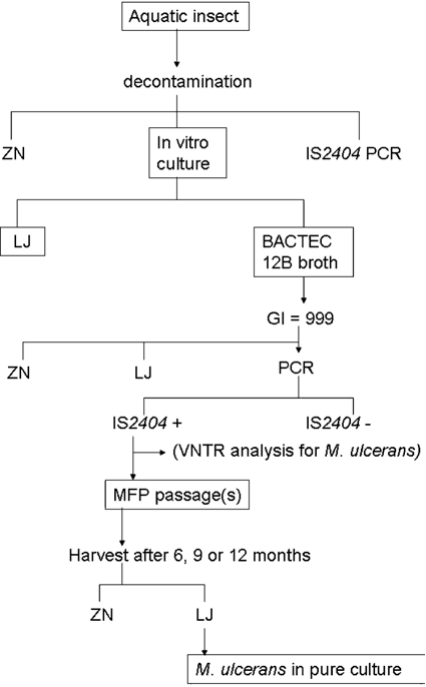
Main steps followed to cultivate *M. ulcerans* in pure culture from an aquatic insect (*Gerris* sp.).

In the present study, the main steps followed to cultivate *M. ulcerans* in pure culture from an aquatic insect are summarized in Fig. 9. Other methods may also be applied such as cultures from salivary glands of wild Naucoridae [9] or aquatic plants [18], or other culture procedures such as the Mycobacteria growth Indicator Tube (MGIT) system [55], or other decontamination methods [27]. The growth of *M. ulcerans* in liquid media can also be confirmed by applying VNTR analysis to the IS*2404* positive liquid cultures to differentiate *M. ulcerans* and other IS*2404* positive mycobacteria [23]. This was not done because the technique had not yet been developed when the present study was undertaken.

In conclusion, for the first time a genetically and phenotypically identified *M. ulcerans* has been isolated in pure culture from an environmental source, reinforcing the concept that the agent of BU is a human pathogen with environmental aquatic niches.
